# How to advance medical education using journal articles? Insight from problem-based learning

**DOI:** 10.3205/zma001569

**Published:** 2022-09-15

**Authors:** Chun-Wai Ma

**Affiliations:** 1 The University of Hong Kong, Li Ka Shing Faculty of Medicine, School of Biomedical Sciences, Hong Kong

**Keywords:** problem-based learning, active learning, student engagement, journal articles, COVID-19

## Letter to the editor

Dear Editor,

Problem-based learning (PBL) is implemented in medical education worldwide. Despite decades of research and discussions, there is no definitive conclusion on the best way for teaching activities of PBL to be optimized [1], [2], [3]. While this issue may be too complex to be fully resolved, is there a basic yet effective method to enhance the benefits of PBL? During the COVID-19 pandemic, some teachers conduct PBL tutorials via video conferencing [4]. Since students spend most of their time at home, how they prepare for PBL tutorials at home significantly influences the learning quality. Is reading journal articles a good option? In the literature, studies that focus specifically on the practicality and effects of using journal articles to assist with PBL in medical curricula are rare.

I assigned medical students in my preclinical PBL groups the task of reading journal articles on biomedical research during the short periods between online PBL tutorials, with each period lasting approximately 5 days on average. The articles that I selected were relevant to the PBL cases but did not provide solutions to the problems in the scenarios. For each group (10 or 11 students), I provided no more than two articles in each period, thus ensuring sufficient reading time. I briefly explained to my students why the articles would be useful in improving their understanding of medical science and preparing for the subsequent tutorials. Nevertheless, the PBL principle of self-directed learning was generally followed. Students’ perception was very positive. They expressed satisfaction and appreciation in their feedback. Better student engagement and performance were also observed during tutorials. The students discussed more comprehensively and raised questions more actively, suggesting that this simple teaching approach helped to promote deep learning, consolidate concepts, and inspire ideas. Further work is planned to assess its impact more extensively in a quantitative manner.

Appropriate inclusion of journal articles in PBL is a method that can be conveniently and rapidly adopted by teachers to reinforce students’ learning experience and broaden their knowledge. In the post-COVID-19 era, it will still be worthwhile to develop teaching strategies that best incorporate journal reading into medical education. Do journal articles strengthen the connection between other didactic materials and student-centered learning? Do journal articles help students appreciate the translation of biomedical discoveries into clinical applications? How should journal articles be used to increase learning motivation? Hopefully, this article will stimulate exploration of these interesting topics. While journal articles may sometimes be considered too traditional, they can be more valuable than expected in supporting pedagogical innovation.

Dr. Chun-Wai Ma

## Competing interests

The author declares that he has no competing interests.
